# A ‘Genome‐First’ Framework for Next‐Generation Bioinputs: From Functional Mining to Rational Synthetic Microbial Communities

**DOI:** 10.1111/1462-2920.70319

**Published:** 2026-05-05

**Authors:** Osiel Silva Gonçalves

**Affiliations:** ^1^ Department of Biological Science, Microbial Eco‐Evolutionary Genomics Group Midwestern Parana State University (Unicentro) Paraná Brazil

**Keywords:** bioinoculants, bioprospecting, genome‐scale metabolic networks, plant‐growth‐promoting bacteria

## Abstract

The demand for sustainable agriculture has shifted bioprospecting towards microbial bioinputs as alternatives to chemical fertilisers and pesticides. Whole‐genome sequencing accelerates the discovery of plant‐growth‐promoting bacteria (PGPB) by enabling the identification of functional genes and the prediction of traits such as nutrient solubilisation, phytohormone production and biocontrol. Traditionally a secondary tool for strain characterisation, genomics has evolved into a ‘genome‐first’ strategy that effectively collapses the phenotypic bottleneck in prospective bioprospecting and the rational design of synthetic microbial communities (SynComs). In this review, we argue for a transition from empirical phenotypic screening towards a genomics‐guided paradigm for the selection of next‐generation bioinputs. This work demonstrates how actionable insights can be gained through the integration of high‐resolution genome mining into discovery pipelines. We explore the application of reverse ecology to infer ecological roles from genomic content and emphasise the critical role of pangenomics in identifying traits linked to host colonisation and niche adaptation. Furthermore, we advocate for biosafety screening as a non‐negotiable prerequisite for bioinoculant development to ensure ecological and clinical safety. Finally, this work proposes that genome‐scale metabolic networks are essential to enable the transition from single‐strain inoculants to the assembly of stable SynComs. This framework establishes a comprehensive, data‐driven approach to predictable interventions in the agricultural bioeconomy.

## Graphical abstract

I propose a ‘genome‐first’ framework to collapse the phenotypic bottleneck in bioinput discovery. By integrating multi‐layered genome mining and reverse ecology, this paradigm enables the rational design of stable synthetic microbial communities for a data‐driven and sustainable agricultural bioeconomy.

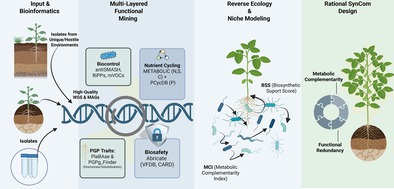

## 1 Introduction

Plant–microbe interactions are fundamental drivers of terrestrial ecosystem productivity, mediating essential functions such as nutrient cycling, pathogen suppression and abiotic stress tolerance (Mendes et al. 2011; Trivedi et al. 2020; Yusuf et al. 2025). The strategic deployment of these microorganisms has catalysed a global surge in the use of bioinputs in sustainable agriculture. Traditionally, the microbial bioinput landscape has been anchored by legacy genera like *Bradyrhizobium*, *Azospirillum* and *Bacillus*, long recognised for their roles in nitrogen nutrition, plant development and pathogen suppression (Lopes et al. 2018; Pelagio‐Flores et al. 2025; Villavicencio‐Vásquez et al. 2025). The success of these classic inoculants has spurred extensive bioprospecting across diverse ecological niches, including the rhizosphere (Philippot et al. 2013), the phyllosphere (Sohrabi et al. 2023) and the endosphere (Hardoim et al. 2015), searching for novel strains with superior functional traits.

Despite the expansion of microbial libraries, the identification of high‐performing strains remains hindered by the phenotypic bottleneck. Conventional screening protocols rely on labour‐intensive in vitro assays, such as colorimetric quantification of phytohormones and acid‐based phosphate solubilisation tests, which require significant infrastructure and time (Gordon and Weber 1951; Schwyn and Neilands 1987; Döbereiner et al. 1995; Nautiyal 1999). Furthermore, these laboratory‐scale assays often fail to reflect the performance of isolates under complex field conditions or within the competitive environment of the plant microbiome (Russ et al. 2023). Consequently, many promising candidates identified in vitro do not achieve the desired efficacy in planta, leading to high attrition rates in the development of commercial bioinputs.

The precipitous decline in the cost of next‐generation sequencing (NGS) and the increasing availability of high‐quality reference genomes have provided a transformative opportunity to overcome these limitations (Goodwin et al. 2016; Satam et al. 2023). By shifting the selection paradigm from phenotypic screening to genomics‐guided discovery, bacterial genomes can now be mined in silico for specific biosynthetic gene clusters (BGCs) and plant‐growth‐promoting (PGP) markers (Levy et al. 2018; Kautsar et al. 2019). This approach enables the targeted prioritisation of strains, streamlining experimental validation and facilitating the rational design of multi‐strain consortia.

In this review, we synthesise current knowledge on the genomics‐driven discovery of beneficial microbial strains. We evaluate the bioinformatics landscape for functional annotation and gene mining and discuss how reverse ecology, the inference of ecological roles from genomic data, can predict complex plant–microbe and microbe–microbe interactions. Finally, we provide a framework for the rational design of synthetic microbial communities (SynComs), highlighting how metabolic complementarity, modelled through genome‐scale metabolic networks, can be leveraged to develop the next generation of robust and efficient bioinputs.

## 2 Genomics‐Driven Discovery of Beneficial Strains

The transition from traditional bioprospecting to genomics‐guided discovery has redefined our understanding of microbial functional potential within the plant holobiont (Vandenkoornhuyse et al. 2015; Lyu et al. 2021). The leverage of high‐quality reference genomes and genomic frameworks enables a shift beyond descriptive cataloguing towards a mechanistic elucidation of the traits that underpin plant–microbe synergies.

### 2.1 The Pangenome as a Driver of Niche Adaptation

**FIGURE 1:**
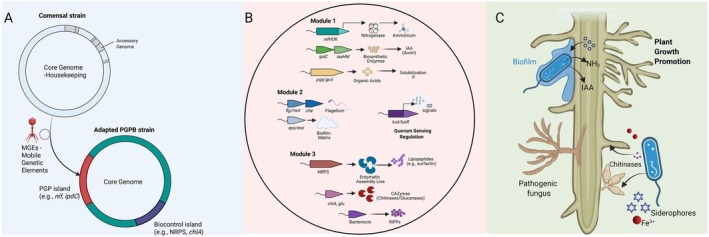
From genome structure to ecological function: the molecular basis of plant‐beneficial interactions. (A) Pangenome‐driven adaptation. Comparative genomics reveals that while related strains share a core genome (blue), niche‐specific traits are often encoded in the accessory genome (coloured segments) acquired via horizontal gene transfer (HGT) mediated by mobile genetic elements (MGEs). This plasticity drives the evolution of specialised plant‐growth‐promoting bacteria (PGPB). (B) The functional genetic toolbox. Within an adapted PGPB, specific gene clusters encode diverse mechanisms. Nutrient acquisition and hormonal modulation involve nitrogen fixation (*nif*), phosphate solubilisation (*pqq*/*gcd*) and auxin biosynthesis (*ipdC*/*iaa*). Fitness traits required for rhizosphere competence include motility (*flg*), chemotaxis (*che*) and biofilm formation (*eps*). The biocontrol arsenal includes biosynthetic gene clusters (BGCs) for lipopeptides (NRPS), secreted CAZymes (e.g., chitinases, *chiA*) and ribosomally synthesised peptides (RiPPs), often regulated by quorum sensing (QS) networks. (C) Rhizosphere interplay. In the soil environment, these genomic traits translate into ecological functions. The PGPB colonises the root surface (rhizoplane), delivering nutrients (NH_3_) and phytohormones (IAA) to enhance plant development, while simultaneously suppressing phytopathogens through the coordinated secretion of lytic enzymes, antimicrobial metabolites and siderophore‐mediated resource competition. Created in BioRender.

The functional landscape of microbial taxa is fundamentally defined by the plasticity of the pangenome, which encompasses the total genetic repertoire of a phylogenetic group. This pangenome is partitioned into the core genome, comprising highly conserved genes essential for vertical inheritance, taxonomic stability and primary metabolism, and the accessory (or flexible) genome, which serves as a dynamic reservoir for niche‐specific adaptations (Medini et al. 2005) (Figure 1A). While the core genome establishes the foundational identity of a taxon, the accessory genome represents the adaptive frontier, dictating the ecological fitness of an isolate within the competitive and chemically complex environment of the rhizosphere (Medini et al. 2005; Tettelin et al. 2005).

Comparative pangenomics across diverse species and strains provides a powerful framework to move beyond empirical, applied microbiology towards a mechanistic understanding of microbial function (Abdul Aziz et al. 2025). The comparative analysis of genomic architectures among isolates with disparate functional outcomes, such as variations in nutrient mobilisation or pathogen antagonism, facilitates the identification of specific accessory genes and genomic islands associated with high‐performance PGP and biocontrol (Polina et al. 2020). This high‐resolution comparative approach reveals that taxonomically near‐identical strains often possess distinct functional trajectories, frequently driven by the acquisition of specialised metabolic modules through horizontal gene transfer (HGT) (Sheinman et al. 2021).

The genus *Stenotrophomonas* exemplifies this dynamic, where a minimal core genome paired with an expansive accessory repertoire of over 15,000 gene families enables broad ecological versatility. The concentration of PGP traits and stress‐tolerance mechanisms within genomic islands and prophages in these strains confirms HGT as a fundamental driver of rhizosphere competence (Zhao et al. 2024). Such niche‐specific adaptation is further reflected in the functional enrichment of PGPB strains according to their primary habitats. Similar patterns of genomic plasticity are observed in the genus *Paenibacillus*, where an open pangenome configuration (*b* = 0.503) highlights ongoing gene acquisition and niche specialisation. In a comparative analysis of 428 high‐quality *Paenibacillus* genomes, the accessory and unique fractions were found to be enriched with genes for environmental adaptation, including mobile genetic elements (MGEs) that facilitate the dissemination of beneficial traits (de Almeida et al. 2026). Comparative analyses of leaf‐associated (LA) and soil‐associated (SA) strains have revealed distinct toolkits for environmental survival; whereas LA strains exhibit enriched pathways for DNA repair and motor chemotaxis to endure phyllosphere stress, SA strains prioritise cell wall‐degrading enzymes and sporulation genes to navigate the soil matrix (Zhen et al. 2023).

Furthermore, the identification of these accessory repertoires is essential for uncovering the underlying molecular drivers of beneficial interactions. Much of the current literature focuses on the broad application of bioinputs; however, genomic mining allows for the discovery of novel genetic clusters that remain invisible in single‐genome analyses. By deciphering how these variable genes contribute to host colonisation and niche occupancy, the field can shift from the retrospective characterisation of isolates to a predictive ‘genome‐first’ strategy, enabling the rational orchestration of microbial functions for sustainable agricultural interventions.

### 2.2 Precision Mining of Functional Pathways

Targeted genome mining allows for the identification of complex metabolic pathways that go beyond simple primary metabolism. In this context, we provide an overview of the fundamental mechanisms of plant‐growth promotion and biocontrol, highlighting the key genetic determinants that serve as blueprints for targeted mining efforts.

#### 2.2.1 Biological Nitrogen Fixation (BNF) and Symbiotic Signalling

Biological nitrogen fixation (BNF) is among the most energetically demanding and evolutionarily significant microbial traits (Olivares et al. 2013). The capacity for diazotrophy is governed by the *nif* regulon, a sophisticated genetic cluster encoding the nitrogenase enzymatic complex. While *nifH* is frequently used as a diagnostic marker, a functional BNF machinery requires the structural genes *nifHDK*, which encode the dinitrogenase reductase and dinitrogenase subunits, alongside an array of accessory genes (*nifENB*, *nifUSVW*) involved in the biosynthesis and maturation of the iron‐molybdenum cofactor (FeMo‐co) (de Nichio et al. 2025) (Figure 1B). In symbiotic lineages, such as the *Rhizobiaceae*, this nitrogenase complex is integrated with *nod*, *nif* and *fix* clusters. The nod genes (e.g., *nodABC*) mediate the synthesis of lipochitooligosaccharide signalling molecules (Nod factors), which are essential for high‐fidelity host recognition and the initiation of root nodule organogenesis (Schultze and Kondorosi 1996, 1998; Mahmud et al. 2020). The precise orchestration of these pathways, often regulated by oxygen‐sensing systems like *fixLJ* and the master regulator *nifA*, ensures that nitrogen fixation is synchronised with the physiological state of the host (Figure 1B).

#### 2.2.2 Phosphate Solubilisation and Nutrient Acquisition

The mobilisation of limiting nutrients, particularly phosphorus (P), is a core functional pillar of PGPB. Genome mining for P‐solubilisation focuses on two main strategies: the mineral solubilisation of inorganic P and the mineralisation of organic P (Santoyo et al. 2026). The primary mechanism for inorganic P‐solubilisation is the secretion of low‐molecular‐weight organic acids (e.g., gluconic, citric, and malic acids), which acidify the rhizosphere and chelate metal cations bound to phosphates (Rengel and Marschner 2005) (Figure 1B). The glucose dehydrogenase (*gdh*) gene, coupled with its essential cofactor pyrroloquinoline quinone (encoded by the *pqqABCDE* operon), represents the metabolic core of gluconic acid production (Cordell and Daley 2022). Furthermore, the genomic fitness of a strain is defined by its ability to sense and transport P under limiting conditions, governed by the pho regulon. This includes the high‐affinity phosphate‐specific transport (Pst) system (*pstABCS* operon) and the two‐component phoR−phoB regulatory system, which modulates the expression of alkaline phosphatases (*phoA*, *phoD*) for organic P mineralisation (Vuppada et al. 2018). These traits are often coupled with the synthesis of high‐affinity siderophores, such as those encoded by the *ent* or *pvd* clusters, which facilitate iron competition in the rhizoplane (Schalk 2025).

#### 2.2.3 Phytohormone Orchestration

Beyond nutrient acquisition, PGPB modulate plant physiology through the synthesis and degradation of phytohormones. While tryptophan‐dependent pathways for Indole‐3‐acetic acid (IAA) production are common, robust genomic inference requires identifying specific conversion enzymes (Figure 1B). These include the indole‐3‐pyruvate (IPyA) pathway, characterised by the *ipdC* gene, or the indole‐3‐acetamide (IAM) pathway (*iaaM* and *iaaH*) (Duca and Glick 2020; Tang et al. 2023). Additionally, the presence of 1‐aminocyclopropane‐1‐carboxylate (ACC) deaminase, encoded by the *acdS* gene, is a critical genomic indicator of a strain's ability to mitigate plant ethylene stress, thereby enhancing root elongation and stress tolerance (Singh et al. 2015).

#### 2.2.4 Rhizosphere Competence and Biofilm Formation

The efficacy of these metabolic pathways is ultimately dependent on the strain's ‘rhizosphere competence’, its ability to navigate the soil matrix and establish a stable association with the host (Compant et al. 2025). Chemotaxis (*che* operons) and motility (*flg*, *mot*, *fli* clusters) are essential for the initial attraction to root exudates. Subsequent colonisation is facilitated by the biosynthesis of extracellular polysaccharides (EPS) and capsules, encoded by *eps*, *exo* and *pel* clusters. These EPS components are central to biofilm formation, creating a protective microenvironment that shields the community from environmental desiccation and pathogen competition, ensuring long‐term functional persistence (Figure 1C).

### 2.3 Genomic Determinants of Biocontrol and Enzymatic Arsenals

Beneficial microorganisms deploy an expansive repertoire of secreted enzymes and specialised metabolites to antagonise phytopathogens and modulate the plant environment. In analogy to the plant cell wall‐degrading enzymes (PCWDEs) used by pathogens to breach host tissues, biocontrol agents utilise carbohydrate‐active enzymes (CAZymes) to target the structural integrity of fungal and oomycete competitors (Kubicek et al. 2014) (Figure 1B). Specifically, the presence of chitinases (e.g., *chiA*, *chiB*), 1,3‐glucanases and proteases constitutes a potent enzymatic arsenal for the degradation of recalcitrant fungal cell wall polymers (Kubicek et al. 2014).

In addition to secreted metabolites and enzymes, the genomic capacity to synthesise microbial volatile organic compounds (VOCs) represents a critical mechanism for long‐distance signalling and biocontrol (Poveda 2021). Key genomic markers include the *alsSD* (acetolactate synthase and decarboxylase) and *butABC* operons, which regulate the conversion of pyruvate into acetoin and 2,3‐butanediol. These volatiles function as essential elicitors of induced systemic resistance (ISR), priming the plant's immune system against a broad spectrum of pathogens without direct contact (Poveda 2021).

Complementing this enzymatic machinery is the genomic potential to synthesise specialised metabolites encoded within biosynthetic gene clusters (BGCs). In the genus *Bacillus*, for instance, non‐ribosomal peptide synthetases (NRPSs) facilitate the modular assembly of potent lipopeptides, such as surfactins, iturins and fengycins, which exhibit broad‐spectrum antimicrobial activity and surfactant properties that aid in biofilm expansion (Saiyam et al. 2024). Similarly, Actinobacteria, particularly members of the *Streptomyces* genus, serve as a prolific reservoir of bioactive compounds, encoding diverse BGCs for the synthesis of antibiotics such as neomycin, which confer a decisive competitive advantage in the polymicrobial landscape of the rhizosphere through antibiosis and nutrient sequestration (Ward and Allenby 2018; van Bergeijk et al. 2020).

Beyond NRPS, the genomic landscape of biocontrol has been expanded by the discovery of ribosomally synthesised and post‐translationally modified peptides (RiPPs), such as bacteriocins. These gene clusters represent a highly specific antimicrobial arsenal that can be precisely mined using tools like BAGEL (de Jong et al. 2006). Furthermore, the efficacy of these functional arsenals is often contingent upon quorum sensing (*luxR*/*luxI* or *lasR*/*lasI* systems) networks, which orchestrate gene expression in response to population density and environmental cues (Fuqua et al. 1996). The localisation of these PGP traits within MGEs and genomic islands further underscores the evolutionary plasticity of the plant microbiome, highlighting the role of HGT in the rapid adaptation of beneficial strains to novel host niches.

## 3 Bioinformatics Tools for Functional Annotation and Gene Mining

The translation of raw genomic data into actionable biological insights depends on a robust bioinformatic foundation. To bypass the ‘phenotypic bottleneck’ effectively, the initial stages of data processing must ensure that the genomic sequence is a faithful representation of the isolate's metabolic potential. In a ‘genome‐first’ framework, the quality of the assembly is not a mere technicality; it is the primary determinant of the accuracy of subsequent functional annotations.

**FIGURE 2:**
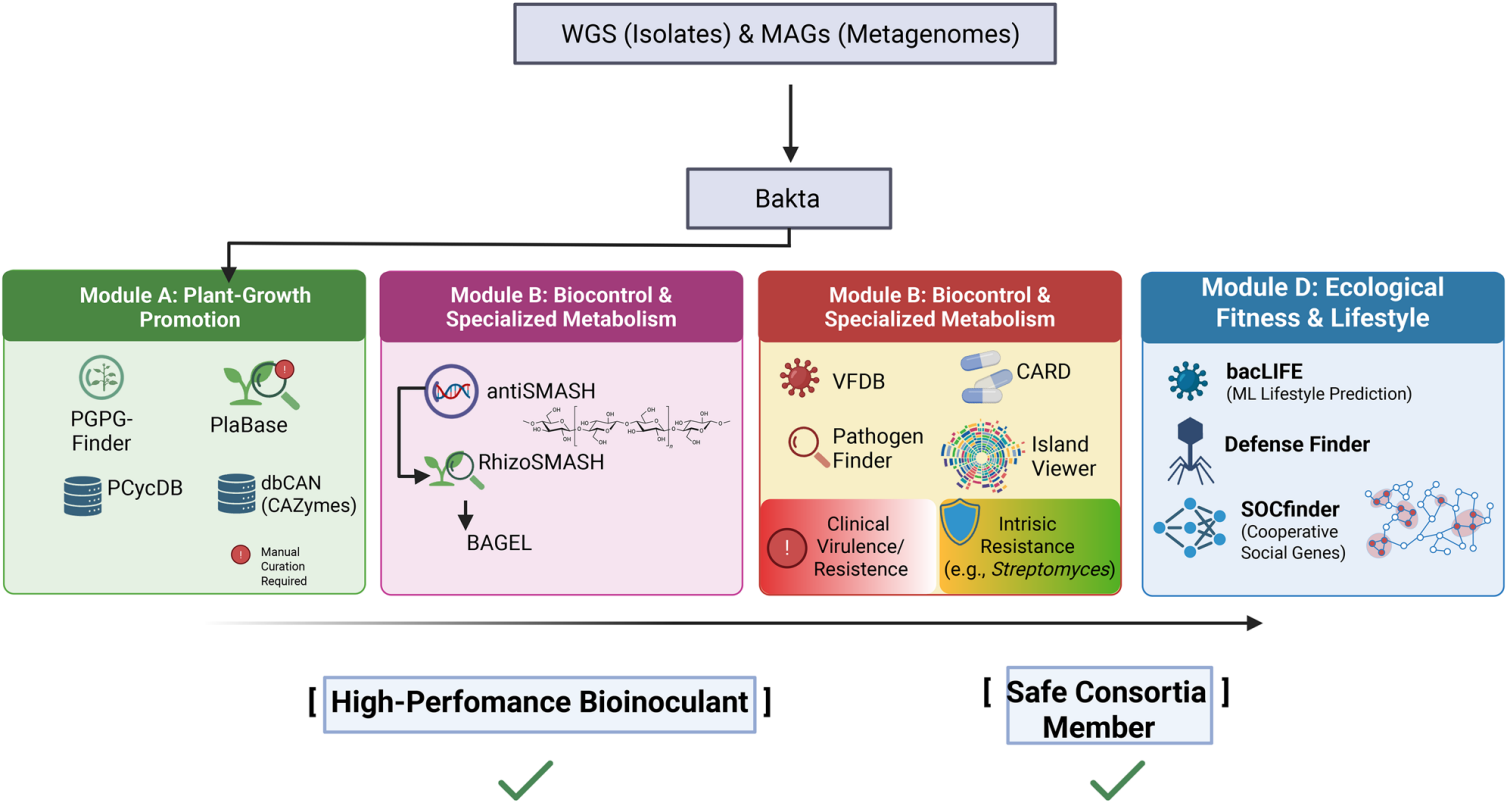
An integrated computational framework for the genomic discovery of plant‐beneficial microbes. The pipeline transitions from primary structural annotation (using Bakta or Prokka) to specialised functional modules. (Top) Genomic and metagenomic inputs are processed to establish the taxonomic and structural scaffold. (Centre) Analysis is bifurcated into four key domains: (i) PGP traits, utilising databases like Plabase; (ii) Biocontrol, employing antiSMASH and RhizoSMASH to map secondary metabolism and root exudate utilisation; and BiG‐SCAPE (iii) Safety, where VFDB and CARD differentiate intrinsic resistance from clinical virulence; and (iv) Fitness, using DefenseFinder and SOCfinder to predict phage resistance and microbial cooperation. (Bottom) Integrative tools such as bacLIFE utilise machine learning to synthesise these multi‐omic data points, providing a high‐confidence prediction of the microbial lifestyle and its potential efficacy as a bioinput in sustainable agriculture. Created in BioRender.

The workflow begins with rigorous quality control of raw data, followed by the selection of an assembly strategy (short‐read, long‐read or hybrid approaches) optimised for the specific genomic complexity of the isolate. The objective is to achieve high structural contiguity, since fragmented assemblies often lead to truncated genes and broken operons, resulting in the underestimation of a strain's true functional repertoire (Thomma et al. 2016). Before proceeding to functional mining, a quantitative assessment of genomic integrity is mandatory. This involves evaluating assembly completeness and contamination levels through the identification of lineage‐specific single‐copy genes. To ensure high‐quality sequences for downstream analysis, we adopted thresholds of > 95% completeness and < 5% contamination. Additionally, genomic continuity was assessed using the N50 metric, which provides a statistical measure of the assembly's fragmentation. Finally, precise phylogenomic identification provides the necessary evolutionary context for functional interpretation. Moving beyond 16S rRNA gene sequences towards whole‐genome‐based taxonomic placement allows for the clear differentiation of beneficial isolates from closely related opportunistic pathogens (Chaumeil et al. 2020). This is robustly achieved using the Genome Taxonomy Database Toolkit (GTDB‐Tk), which assigns taxonomy by placing assemblies into the GTDB reference tree based on the presence of multiple concatenated single‐copy marker genes. This approach, complemented by the calculation of average nucleotide identity (ANI) values, ensures a high‐resolution classification that overcomes the limited resolution of individual marker genes (Figure 2).

### 3.1 Standardising Genome Annotation

High‐throughput annotation is the cornerstone of microbial characterisation. While Prokka (Seemann 2014) and RAST (Aziz et al. 2008) have served as established benchmarks for years, newer tools like Bakta have emerged to provide superior nomenclature consistency and faster processing times, particularly for large‐scale genomic studies (Schwengers et al. 2021). These pipelines provide the structural scaffold, identifying protein‐coding genes, tRNAs and rRNAs upon which more specialised functional mining is built.

### 3.2 Mining for Growth Promotion and Biocontrol Traits

The identification of PGP traits requires specialised algorithms that go beyond general metabolic annotation.

#### 3.2.1 PGP‐Specific Tools

Until recently, the prediction of PGP traits relied heavily on manual queries within general‐purpose databases to identify candidate genes. However, these generalist resources often lack the ecological context required for high‐resolution PGP mining. To address this gap, specialised web resources like PLaBAse have been developed (Patz et al. 2021), providing a dedicated infrastructure for screening plant‐associated bacteria. This platform integrates the PLaBA‐db (a database of over 5000 plant‐associated genomes), PIFAR‐Pred for identifying bacterial plant‐association markers and PGPT‐Pred for the prediction of specific PGP traits (Figure 2).

While the PLaBAse web server is a valuable resource for individual genome queries, it can yield a high number of potential hits that may not be directly involved in the desired functional activity. This necessitates rigorous curation to confirm the presence of complete, syntenic gene clusters rather than isolated gene fragments. To ensure computational efficiency during large‐scale bioprospecting, specialised pipelines like PGPg‐finder (Pellegrinetti et al. 2024) facilitate the streamlined analysis of hundreds of genomes and metagenomes, particularly when executed locally to bypass the limitations of web‐based platforms.

#### 3.2.2 Secondary Metabolism and Biocontrol

The discovery of antimicrobial arsenals and metabolic specialised modules is primarily driven by antiSMASH, the gold standard for identifying biosynthetic gene clusters (BGCs) (Blin et al. 2019). Recent expansions in the antiSMASH ecosystem have introduced highly specialised tools that address specific ecological requirements of the plant microbiome. RhizoSMASH enables the prediction of gene clusters involved in the catabolism of root exudates, which is a key trait for establishing rhizosphere competence and niche occupancy (Li et al. 2025). Complementarily, epsSMASH facilitates the identification of known and novel exopolysaccharide (exoPS) BGCs (Daugberg et al. 2025), providing insights into the genetic basis of biofilm formation and environmental resilience (Figure 2).

To manage the vast output of these predictive tools, the integration of BiG‐SCAPE (Biosynthetic Gene Similarity Clustering and Prospecting Engine) is essential (Navarro‐Munoz et al. 2018). BiG‐SCAPE constructs sequence similarity networks that group BGCs into gene cluster families (GCFs), allowing for large‐scale comparative analyses across hundreds of genomes. By cross‐referencing these clusters with the MIBiG (Minimum Information about a Biosynthetic Gene cluster) repository (Kautsar et al. 2019), it enables the effective de‐replication of known pathways and prioritises truly novel biosynthetic potential for further characterisation.

Beyond non‐ribosomal peptides and polyketides, specialised resources like BAGEL focus on the identification of ribosomally synthesised and post‐translationally modified peptides (RiPPs), such as bacteriocins (de Jong et al. 2006). Furthermore, the mVOC 4.0 database (Lemfack et al. 2014) serves as a critical resource for the study of microbial volatiles, cataloguing thousands of volatile organic compounds (VOCs) and their biological functions. This database enables the linkage of genomic signatures, such as the *alsSD* or *butABC* operons, to specific volatile‐mediated outcomes, including the ISR and long‐distance inter‐kingdom signalling. Additionally, dbCAN remains essential for the automated annotation of CAZymes such as chitinases and glucanases (Zhang et al. 2018).

### 3.3 Lifestyle Inference and Multi‐Omics Integration

Predicting how an isolate will behave in the rhizosphere requires an understanding of its ecological ‘lifestyle’.

#### 3.3.1 Lifestyle Assessment

The bacLIFE workflow represents a state‐of‐the‐art approach for large‐scale comparative genomics, utilising machine learning to predict bacterial lifestyles (e.g., pathogenic vs. beneficial) (Guerrero‐Egido et al. 2024). This is particularly crucial for genera like *Pseudomonas*, *Enterobacter* and *Burkholderia*, which contain both biocontrol and PGP agents and potent (phyto)pathogens (Guerrero‐Egido et al. 2024) (Figure 2).

#### 3.3.2 Ecological Stability and Cooperation

Viral predation represents a pervasive biotic filter in the rhizosphere, as bacteriophages are the most abundant biological entities in soil ecosystems and exert significant top‐down pressure on microbial populations (Yang et al. 2023; Wang et al. 2024). Consequently, the ecological resilience of a candidate isolate can be assessed via DefenseFinder, which identifies specialised phage‐defence systems, for example, CRISPR‐Cas, restriction‐modification modules and abortive infection systems (Doron et al. 2018). These mechanisms are not only essential for survival against viral attack but also serve as key drivers of HGT and genomic diversification in the soil matrix (Figure 2). Complementing this, identifying the genomic basis of social interactions is vital for robust community assembly. SOCfinder enables the detection of ‘cooperative’ genes, including those encoding secreted public goods and extracellular proteins (Belcher et al. 2023). Prioritising these social determinants enables a more accurate prediction of strain performance within SynComs, shifting the focus from individual fitness to collective metabolic stability and long‐term persistence in the rhizosphere.

#### 3.3.3 Metabolic Cycling

The transition towards a ‘genome‐first’ strategy requires tools capable of scaling functional annotation from individual isolates to complex microbial communities or large‐scale genomic datasets. The METABOLIC (METabolic And BiogeOchemistry anaLyses In miCrobes) pipeline represents a sophisticated framework for this high‐throughput profiling, enabling the rapid reconstruction of metabolic networks across hundreds of genomes or metagenome‐assembled genomes (MAGs) (Zhou et al. 2022) (Figure 2). By integrating diverse hidden Markov model (HMM) databases, including KEGG, TIGRfam, dbCAN2 and MEROPS, METABOLIC bypasses the limitations of single‐database queries. Crucially, it incorporates a protein motif validation step based on prior biochemical evidence, ensuring that identified pathways for carbon, nitrogen, sulphur and iron cycling are not merely present but biologically plausible.

However, while METABOLIC provides an expansive overview of major elemental cycles, it lacks a high‐resolution dedicated module for the Phosphorus (P) cycle, a critical oversight given that P‐availability is a primary limiting factor in global agricultural productivity (Santoyo et al. 2026). To achieve a comprehensive nutritional and functional profile for PGPB, it is essential to supplement this analysis with specialised resources such as PCycDB (Phosphorus Cycling Database) (Zeng et al. 2022). The integration of PCycDB allows for the precise mapping of genes involved in inorganic phosphate solubilisation, organic phosphorus mineralisation and high‐affinity transport systems.

### 3.4 Safety Profiling: Virulence and Resistance

A critical, often overlooked step in the development of bioinoculants is the early exclusion of undesirable traits that could pose risks to human health or ecological stability. In a ‘genome‐first’ framework, biosafety screening is a non‐negotiable prerequisite for regulatory approval and field application (Figure 2).

#### 3.4.1 Mass Screening of Virulence and Resistance Determinants

The magnitude of contemporary bioprospecting demands specialised pipelines capable of high‐throughput contig screening to ensure rapid and accurate functional annotation. Pipelines such as Abricate (https://github.com/tseemann/abricate) represent a standard in the field, allowing for the simultaneous query of a single genome against multiple curated databases, including NCBI, CARD, ResFinder and ARG‐ANNOT for antimicrobial resistance (AMR), as well as the Virulence Factor Database (VFDB), Ecoli_VF and VICTORS for virulence factors.

#### 3.4.2 Distinguishing Pathogenicity From Functional Self‐Protection

Pathogenicity screening must be interpreted with biological nuance. While tools like PathogenFinder 2.0 (Ferrer Florensa et al. 2025) and IslandViewer are instrumental in detecting genomic islands associated with virulence, it is imperative to distinguish between clinical risk and intrinsic metabolic mechanisms (Dhillon et al. 2013). To achieve this distinction, pathogenicity screening should be complemented by a comparative genomic approach. Tools such as VFDB and VICTOR allow for the identification of specific virulence determinants (e.g., specialised secretion systems like T3SS or T6SS and toxins) that are often absent in purely environmental isolates. Furthermore, the use of deep‐learning‐based tools like DeepARG (Arango‐Argoty et al. 2018) can help differentiate between intrinsic resistance mechanisms, common in soil microbes for ecological competition, and mobile resistance genes that pose a clinical threat. By integrating these insights with metabolic reconstruction, we can clarify whether a genomic trait serves for host colonisation and plant protection or contributes to clinical pathogenicity.

#### 3.4.3 Ensuring Regulatory and Ecological Compliance

By utilising the Comprehensive Antibiotic Resistance Database (CARD) (Alcock et al. 2020) and ResFinder (Bortolaia et al. 2020) as part of a consolidated screening pipeline, the ‘genome‐first’ strategy ensures that candidate strains do not harbour the ‘resistome’ that could contribute to the global crisis of multi‐drug resistance (Salam et al. 2023). For instance, in prolific secondary metabolite producers such as *Streptomyces*, antibiotic resistance genes are frequently co‐localised within the BGC. In this context, these genes serve as essential self‐protection mechanisms for the producer strain rather than indicators of pathogenic potential (Ogawara 2016). Deciphering the genomic context, specifically whether resistance is intrinsic or associated with highly mobile elements, is vital for the accurate risk assessment of prospective bioinputs.

The assessment of genomic risk is conducted by analysing the flanking regions of identified ARGs and virulence factors. By utilising tools such as geNomad, MobileOG‐db, ISFinder (Siguier et al. 2006; Brown et al. 2022; Camargo et al. 2024), we can determine if these features are located on MGEs. Furthermore, a comparative synteny analysis against type‐strain genomes allows us to distinguish between ancestral intrinsic mechanisms, which are vertically inherited and often part of the core genome, and acquired elements that pose a significant biosafety concern for environmental release.

The computational toolkit for identifying plant‐growth‐promoting traits and ensuring biosafety is summarised in Table 1.

**TABLE 1:** The Genomic toolbox for plant‐growth‐promoting (PGP) traits discovery.

Category	Recommended tools	Primary function
Annotation	Bakta, Prokka	Structural and functional genome annotation
PGP traits	PlaBase, PGPT‐Pred, PGPg‐finder	Detection of nutrient mobilisation and hormone pathways
Carbohydrate‐active enzymes (CAZymes)	antiSMASH, RhizoSMASH, BiG‐SCAPE	BGC mining for antibiotics and exudate catabolism
CAZymes	dbCAN3	Profiling of lytic enzymes (chitinases, cellulases)
Biosafety and risk assessment	VFDB, CARD, Abricate	Screening for virulence and antibiotic resistance
Lifestyle	bacLIFE	ML‐based prediction of pathogenic vs. beneficial roles
Metabolic and biogeochemical cycling	METABOLIC, PCycDB	Biogeochemical cycling of N, P, K and Fe
Ecology	DefenseFinder, SOCfinder	Phage resistance and microbial cooperation profiling

## 4 Reverse Ecology: From Genomes to Ecological Function

The emergence of reverse ecology has redefined the microbial genome as a historical record of selective pressures, providing a bridge between high‐throughput genetic data and large‐scale ecological inference (Levy and Borenstein 2012). Central to this framework is the premise that an organism's metabolic repertoire is inextricably linked to the biochemical landscape it inhabits. The reconstruction of genome‐scale metabolic models from sequence data enables the characterisation of a habitat's biochemical landscape without the need for prior phenotypic observation.

**FIGURE 3:**
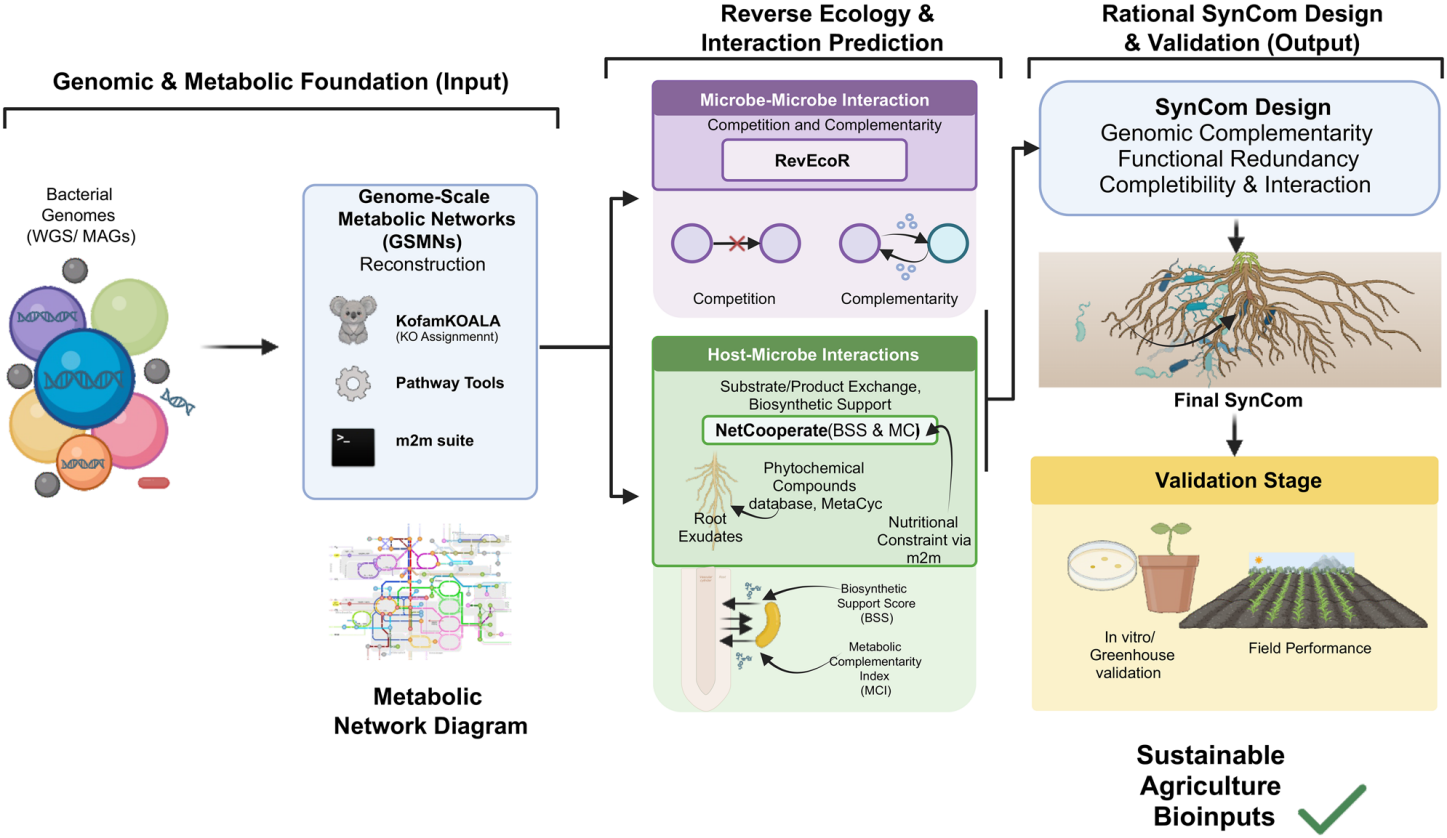
A reverse ecology‐driven pipeline for rational SynCom design. The process initiates with the reconstruction of Genome‐Scale Metabolic Networks (GSMNs) from whole‐genome sequences (WGS) or metagenome‐assembled genomes (MAGs), employing tools like KofamKOALA, PathwayTools and the m2m suite. This foundational metabolic blueprint then fuels a reverse ecology framework for predicting ecological interactions. (Left‐Centre) Microbe–microbe interactions are quantified using RevEcoR to assess competition and complementarity within potential SynCom members. Simultaneously, host–microbe interactions are predicted via NetCoo‐perate (measuring BSS and MCI), with metabolic constraints refined by root exudate profiles (Phytochemical Compounds Database). (Right) These integrated genomic insights inform the rational design of SynComs, prioritising genomic complementarity, functional redundancy and optimal interaction dynamics. The designed communities then proceed to rigorous experimental validation across in vitro, greenhouse, and field settings, culminating in the development of robust, next‐generation bioinputs for sustainable agriculture.

The transition from genomic sequence to ecological insight begins with high‐fidelity functional annotation, typically involving tools such as KofamKOALA to assign KEGG Orthology (KO) identifiers (Aramaki et al. 2020) (Figure 3). These identifiers are then utilised to map the metabolic ‘seed set’, defined as the collection of essential compounds that an organism cannot synthesise and must acquire from its environment (Levy and Borenstein 2012). Building upon this metabolic reconstruction, graph‐theory‐based algorithms allow for the prediction of complex biotic interactions. Indices of metabolic competition and cooperation can be calculated through RevEcoR and the Cooperation Index package, allowing for the in silico evaluation of interaction dynamics and functional stability (Cao et al. 2016). For instance, high metabolic complementarity suggests potential syntrophy, where cross‐feeding allows a consortium to thrive in environments where individual strains would fail.

When applied to host–microbe interactions, this framework employs specialised tools like NetCooperate to gain insights into the metabolic synergy between plants and their associated microbiota (Levy et al. 2015). The quantification of the biosynthetic support score (BSS) and the metabolic complementarity index (MCI) enables the prediction of the degree to which a plant host provides the requisite metabolic precursors for successful microbial colonisation (Figure 3). These interactions are further refined by cross‐referencing results with the Phytochemical Compounds Database and MetaCyc (Caspi et al. 2020), ensuring that the predicted exchanges align with the known secondary metabolism of the host plant. Such metrics are vital for identifying strains that are not only beneficial but also ecologically compatible with specific crops.

The practical efficacy of this approach is exemplified by its application in designing a bacterial consortium specifically tailored for soybean ( *Glycine max* ). In this model, the reverse ecology framework identified a core assembly of *Paenibacillus polymyxa* , *Methylobacterium brachiatum* and *Enterobacter* sp. based on their high synergistic potential and low metabolic competitiveness (Gonçalves et al. 2024). The in silico prediction of complementary metabolic profiles, suggesting minimal resource overlap and potential mutualism, was subsequently validated through in vitro stability assays and greenhouse trials. These experiments confirmed that the genome‐led selection of strains not only maintained community stability without inhibitory effects but also significantly enhanced soybean development in the initial stage (Gonçalves et al. 2024).

However, despite its transformative potential, the field of reverse ecology is currently hindered by significant technical and experimental bottlenecks. Many foundational tools remain outdated or were originally developed for small‐scale datasets, struggling to maintain pace with the volume of data generated by modern genomic and metagenomic studies (Figure 3). Furthermore, there is a critical ‘validation gap’ between computational scores and biological reality.

## 5 Rational Design of Synthetic Microbial Communities (SynComs)

The limitations of single‐strain inoculants, often characterised by inconsistent field performance and poor survival in competitive soil matrices, have catalysed a shift towards the development of SynComs (Figure 3). These next‐generation bioinputs replicate the robustness of natural microbiomes while maintaining a manageable level of complexity. The transition from random strain mixing to rational design is now driven by genome‐scale metabolic networks (GSMNs), which serve as comprehensive computational models of the entire metabolic potential of an organism (Terzer et al. 2009; Cooke et al. 2024). The reconstruction of GSMNs enables the elucidation of the intricate metabolic interdependencies between microorganisms and their host plants, ensuring that the resulting community is defined by genomic complementarity and functional redundancy.

### 5.1 High‐Throughput Metabolic Reconstruction

The assembly of a high‐performance SynCom begins with the automated reconstruction of non‐curated metabolic networks, a process streamlined by tools such as the metage2metabo (m2m) suite (Belcour et al. 2020). The utilisation of high‐quality genomic data (WGS or MAGs) allows for the deployment of PathwayTools to encapsulate the metabolic reactions and pathways encoded within each genome (Karp et al. 2002) (Figure 3). In large‐scale genomic analyses, the mpwt (multiprocessing pathway tools) utility is employed to orchestrate a PathoLogic environment for multiple inputs simultaneously, followed by the m2m recon command to build the foundational networks. This integrative approach allows for the assessment of the collective metabolic potential (the ‘metabolic scope’) of a community, revealing how individual strains contribute to overall ecosystem functions (Belcour et al. 2020).

### 5.2 Incorporating Host‐Specific Constraints

To move beyond theoretical metabolic potential, GSMNs must be refined by incorporating the metabolic constraints of the host plant and the nutritional landscape of the rhizosphere. By utilising root exudate‐mimicking growth media as a ‘seed set’, it is possible to predict metabolic outputs that are restricted to those metabolites realistically producible within the specific chemical niche of the root zone. This step is pivotal for reducing the search space of predicted interactions and focusing on the actual metabolic niches available to the microbes. As demonstrated in recent genomic‐led studies (Gonçalves et al. 2023), this framework facilitates the identification of ‘core’ community members that provide essential services, while simultaneously identifying ‘accessory’ strains that enhance the consortium's resilience to environmental fluctuations.

### 5.3 Balancing Complementarity and Redundancy

Ultimately, the rational design of SynComs hinges on a strategic balance between metabolic complementarity, where strains occupy distinct niches or engage in syntrophy, and functional redundancy, which ensures that the loss of a single strain does not lead to the collapse of the community's beneficial traits. This balancing act is fundamentally underpinned by the high‐resolution functional characterisation detailed in previous sections (see Section 3), which is integrated into the reverse ecology framework to predict interactions. By leveraging GSMNs and tools like m2m in the context of (meta)genomics, the field is moving towards a predictable orchestration of microbial functions. This strategy not only accelerates the discovery of effective bioinoculants but also ensures their long‐term stability and compatibility.

## 6 Outlook

The global shift towards sustainable agriculture necessitates a departure from traditional, empirical bioprospecting. As outlined in this review, the transition to a ‘genome‐first’ strategy represents a fundamental shift in how we prioritise microbial candidates. By leveraging WGS at the earliest stages, we can collapse the ‘phenotypic bottleneck’, moving directly from sequence to the prediction of complex traits like nutrient solubilisation and biocontrol. This approach is particularly transformative when applied to isolates from underexplored or hostile environments, such as the rhizosphere, phyllosphere and endosphere of plants adapted to extreme salinity, drought or nutrient deficiency (Eshel et al. 2021; Camargo et al. 2023). In these ecological frontiers, this framework sheds light on taxonomically novel groups and ‘genomic dark matter’, significantly increasing the probability of discovering unconventional metabolic pathways and high‐performance traits that remain elusive under standard laboratory screenings.

The effectiveness of this framework hinges on our ability to integrate comparative genomics with reverse ecology. While the core genome establishes taxonomic identity, the accessory genome harbours the determinants of niche adaptation and rhizosphere competence. Understanding these genomic ‘islands’ is crucial for ensuring that selected strains are capable of robust host colonisation. Furthermore, by employing GSMNs and tools like metage2metabo, we can predict microbial interaction dynamics in silico. This allows for the rational design of SynComs.

Crucially, the ‘genome‐first’ framework also serves as a critical biosafety gatekeeper, ensuring the early exclusion of virulence and antibiotic resistance genes. A final transformative frontier is the application of machine learning (ML) to decode the universal molecular rulebook of host association. By identifying conserved functional motifs, ML‐driven pipelines allow researchers to recognise high‐performance functional groups even in poorly characterised or taxonomically distant lineages. Ultimately, this synergy between environmental exploration and genomic intelligence will enable the identification of candidate bioinoculants based on their true functional potential rather than their phylogenetic proximity to known PGPB, paving the way for targeted, data‐driven interventions in the agricultural bioeconomy.

## Author Contributions

**Osiel Silva Gonçalves:** conceptualization, investigation, funding acquisition, visualization, writing – review and editing, software, data curation, supervision, writing – original draft.

## Conflicts of Interest

The author declares no conflicts of interest.

## Data Availability

Data sharing not applicable to this article as no datasets were generated or analysed during the current study.
